# *Laetiporus sulphureus* Mycelial Extract Promotes Melanogenesis via p38 MAPK-Mediated Activation of β-Catenin/MITF and Rho Family Proteins in B16F10 Cells

**DOI:** 10.4014/jmb.2510.10055

**Published:** 2025-12-09

**Authors:** So Jung Park, Hyeok Jin Choi, Jeong Won Choi, Gyeong Eun Im, Youngki Park, Kyoung Tae Lee, Jin Boo Jeong

**Affiliations:** 1 Department of Forest Science, Gyeongkuk National University, Andong 36729, Republic of Korea; 2 Department of Forest Bioresources, Division of Forest Microbiology, National Institute of Forest Science, Suwon 16631, Republic of Korea

**Keywords:** *Laetiporus sulphureus*, melanogenesis, extracellular melanin, intracellular melanin

## Abstract

*Laetiporus sulphureus* mycelial extract (LSM) has shown potential as a pigmentation modulator. This study investigated the effects of LSM on melanogenesis in B16F10 cells. LSM significantly increased both extracellular and intracellular melanin content. It also upregulated tyrosinase protein expression and activity, along with MITF and β-catenin levels. LSM activated ERK1/2, p38, and JNK phosphorylation; however, only p38 inhibition suppressed LSM-induced melanin synthesis and the expression of melanogenesis-related proteins. Moreover, LSM increased the protein levels of Rho family GTPases (Cdc42, Rac1, RhoA), which was also blocked by p38 inhibition. These findings suggest that LSM promotes melanogenesis through p38 MAPK-dependent activation of β-catenin/MITF/tyrosinase axis and Rho GTPases, highlighting its potential as a natural pigmentation enhancer.

## Introduction

Skin and hair pigmentation depends on the quantity and quality of melanin (eumelanin/pheomelanin), the maturation of melanosomes, and the efficiency of melanin transfer from melanocytes to keratinocytes. Beyond cosmetic outcomes, melanin provides frontline photoprotection by absorbing ultraviolet radiation and mitigating oxidative stress, thereby reducing photoaging and photocarcinogenesis [1]. Dysregulation of melanocyte function underlies hyper- and hypopigmentary disorders; in hypopigmentation (*e.g.*, vitiligo), therapeutic strategies increasingly aim to restore melanocyte function and stimulate melanogenesis to achieve repigmentation [2, 3]. Melanogenesis is orchestrated within melanosomes by the rate-limiting enzyme tyrosinase and the tyrosinase-related protein-1/2 under transcriptional control of microphthalmia-associated transcription factor (MITF) [4]. The transport and export of melanosomes also determine pigmentation output. Melanocytes acquire a polarized, dendritic architecture to traffic mature melanosomes from the perinuclear region to dendrite tips for transfer to neighboring keratinocytes [5]. Small Rho family GTPases (Rac1, Cdc42, RhoA) remodel the actin cytoskeleton and regulate dendrite formation and melanosome dynamics; Rac1, in particular, is necessary for α-MSH- and UV-induced dendricity in melanocytic cells [6, 7].

Despite recent advances in pharmacological and phototherapeutic approaches for treating hypopigmentation disorders, current modalities such as topical corticosteroids, calcineurin inhibitors, Janus kinase inhibitors, and narrowband UVB phototherapy often present limitations including local and systemic side effects, risks associated with prolonged use, and limited long-term efficacy [8]. These concerns underscore the pressing need for alternative or adjunctive strategies that are both effective and safe for chronic use. In this context, natural compounds derived from medicinal plants and fungi have garnered increasing interest as promising pigmentation modulators due to their multi-target mechanisms, low toxicity profiles, and potential to restore melanocyte function and dendricity without adverse effects [9].

Among various natural candidates, *Laetiporus sulphureus* has attracted growing attention for its diverse pharmacological properties. Widely distributed in temperate regions, *L. sulphureus* produces bioactive metabolites with antioxidant [10], anti-inflammatory [11], antimicrobial [10], anticancer [12], and hepatoprotective activities [13] from both its fruiting bodies and cultured mycelia. Furthermore, recent studies have shown that the ethanol extract of *L. sulphureus* significantly inhibits melanogenesis in zebrafish embryos in a dose-dependent manner without inducing observable toxicity up to approximately 500 μg/ml [14]. These findings support the potential of *L. sulphureus* as a safe, multifunctional natural agent for modulating pigmentation pathways.

Interestingly, in contrast to previous reports indicating anti-melanogenic effects of *L. sulphureus* [14], our preliminary results revealed that its mycelial extract significantly promoted melanin production in B16F10 cells. Therefore, this study aimed to investigate the underlying mechanisms of this pro-melanogenic activity, with a focus on the involvement of the p38/β-catenin/MITF signaling and p38/Rho GTPases pathways. Our findings seek to elucidate a novel functional potential of *L. sulphureus* as a pigmentation enhancer with therapeutic implications for hypopigmentation disorders.

## Materials and Methods

### Reagents and Antibodies

The following chemicals were sourced from Sigma-Aldrich (USA): PD98059 [extracellular signal-regulated protein kinase 1 and 2 (ERK1/2) inhibitor; cat. no. 513000], SB203580 (p38 inhibitor; cat. no. 58307), and SP600125 [c-Jun N-terminal kinase (JNK) inhibitor; cat. no. 55567)]. Primary antibodies were used as follows: ERK1/2 (cat. no. 9102), phospho-ERK1/2 (cat. no. 4377), p38 (cat. no. 9212), phospho-p38 (cat. no. 4511), JNK (cat. no. 9258), phospho-JNK (cat. no. 9251), β-catenin (cat. no. 9562), Cell division cycle 42 (Cdc42, cat. no. 2462), Ras-related C3 botulinum toxin substrate 1 (Rac 1, cat. no. 4651), Ras homolog family member A (RhoA, cat. no. 2117), and β-actin (5125) (all from Cell Signaling Technology, USA); tyrosinase (cat. no. sc-20035) from Santa Cruz Biotechnology (USA); and microphthalmia-associated transcription factor (MITF; cat. no. MAB3747) from Merck Millipore (USA). HRP-conjugated secondary antibodies such as anti-rabbit IgG (cat. no. 7074) and anti-mouse IgG (cat. no. 7076) were from Cell Signaling Technology. Unless otherwise specified, reagents were analytical grade and used according to the manufacturer’s instructions.

### Sample Preparation

**Fig. 1:**
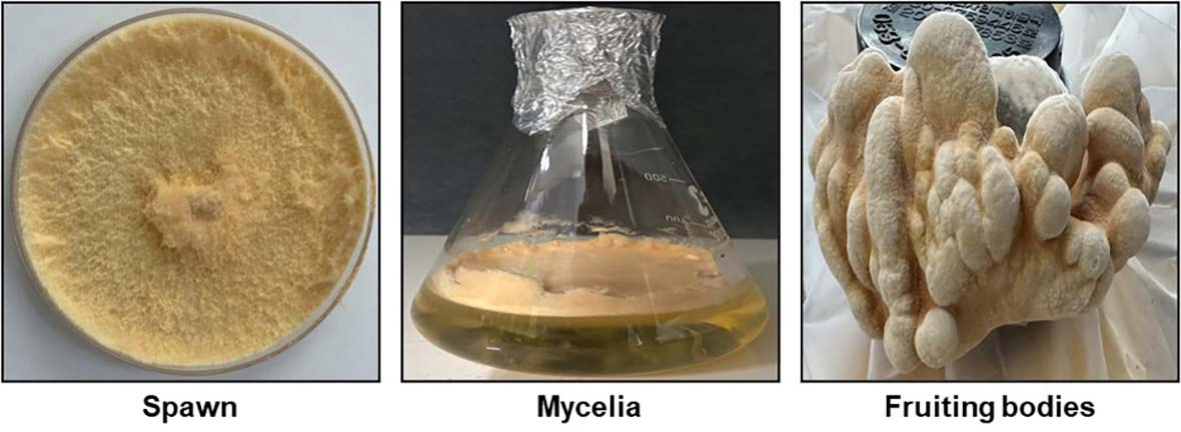
Morphological features of *Laetiporus sulphureus* at different developmental stages. (**A**) Spawn cultured on potato dextrose agar (PDA) medium at 25°C for 15 days. (**B**) Mycelia produced in potato dextrose broth (PDB) medium after 30 days of incubation at 25°C. (**C**) Fruiting bodies cultivated on a substrate mixture of cottonseed hull, wheat bran, and oak sawdust under controlled environmental conditions.

The *L. sulphureus* strain NIFoS 1750 was obtained from the Korea Forest Bioresource Conservation, National Institute of Forest Science (Republic of Korea). The strain was maintained on potato dextrose agar (PDA; Difco, USA) at 25°C for 15 days and used as spawn (Fig. 1A). For bulk mycelial cultivation, potato dextrose broth (PDB; Difco) was inoculated in 500 mL Erlenmeyer flasks and incubated at 25°C for 30 days under static conditions. After cultivation, the liquid cultures were filtered to obtain the mycelial biomass (Fig. 1B). Fruiting bodies were cultivated using a substrate mixture composed of cottonseed hull and wheat bran (1:1, w/w) combined with oak sawdust (7:3, w/w). The substrate was inoculated with spawn and incubated at 25°C for 40 days. Primordia formation was induced by incubation at 4°C for 24 h, followed by cultivation at 21°C for 50 days to obtain mature fruiting bodies (Fig. 1C). The harvested mycelia and fruiting bodies were frozen at -70 °C, freeze-dried, and pulverized into fine powder. The powdered samples were extracted by immersion in 70% ethanol at a volume three times their dry weight. Extraction was repeated three times using an ultrasonic extractor for 1 h each cycle. The combined extracts were filtered and concentrated under reduced pressure to obtain the final samples for analysis. For subsequent experiments, the *L. sulphureus* mycelial extract and fruiting body extract were referred to as LSM and LSFB, respectively.

### Cell Culture

The murine melanoma cell line B16F10 (cat. no. CRL-6475) was obtained from the American Type Culture Collection (USA) and expanded under routine conditions. Cells were maintained in DMEM/F-12 supplemented with 10% heat-inactivated fetal bovine serum, 100 U/ml penicillin, and 100 μg/ml streptomycin in a humidified incubator at 37°C with 5% CO_2_. The culture medium was refreshed every 48-72 h, and cells were re-seeded at ~70-80% confluence using 0.05% trypsin-EDTA. Human epidermal melanocytes (moderately pigmented; HEMn-MP, cat. no. C1025C) were obtained from Life Technologies (USA). Cells were maintained in 254 medium (Life Technologies) containing 1% human melanocyte growth supplement and 1% penicillin-streptomycin, and incubated at 37°C in a humidified atmosphere of 5% CO_2_. All experiments were conducted with cultures at ≤15 passages to minimize phenotypic drift and ensure reproducibility.

### Cell Viability Assay

Cell viability was evaluated using an MTT colorimetric assay. B16F10 melanoma cells were seeded in 96-well plates at a density of 1×10^4^ cells/well in DMEM supplemented with 10% FBS and allowed to attach overnight. The cells were then treated with LSM (12.5, 25, and 50 μg/ml) for 48 h. At the end of the treatment period, the culture medium was replaced with fresh medium containing 0.5 mg/ml MTT, and the plates were incubated for an additional 3 h at 37°C in a humidified CO_2_ incubator. After incubation, the supernatant was carefully removed, and the resulting formazan crystals were dissolved in 100 μl of DMSO per well. The absorbance was measured using a microplate reader at 570 nm. Cell viability was calculated as a percentage of the absorbance of the untreated control group.

### Measurement of Extracellular Melanin (ECM) and Intracellular Melanin (ICM)

B16F10 or HEMn-MP cells were seeded in 6-well plates and treated with the sample for 48 h. Following treatment, the culture medium was collected and centrifuged at 12,000 ×*g* for 5 min to remove any cellular debris. The supernatant was transferred to a fresh tube, and the optical density was measured at 405 nm using a UV/visible spectrophotometer (SpectraMax M2, Molecular Devices, USA) to quantify ECM levels. After removing the culture medium, the cells were washed twice with cold phosphate-buffered saline (PBS) and harvested by trypsinization. The collected cells were pelleted by centrifugation (12,000 ×*g*, 5 min) and lysed in 1 N NaOH containing 10% DMSO. The lysates were incubated at 80°C for 1 h to solubilize melanin. The absorbance of the solubilized samples was then measured at 405 nm with a UV/visible spectrophotometer (SpectraMax M2, Molecular Devices) to quantify ICM level.

### Measurement of Cellular Tyrosinase Activity

B16F10 cells were seeded in 6-well plates and treated with the sample for 48 h. After treatment, cells were washed twice with ice-cold PBS and harvested by scraping. The collected cells were lysed in 50 mM sodium phosphate buffer (pH 6.8) containing 1% Triton X-100 and 0.1 mM PMSF at 4°C for 30 min to ensure complete lysis. The lysates were clarified by centrifugation at 12,000 ×*g* for 15 min at 4°C, and the supernatants were collected as enzyme extracts. Protein concentration was determined by the bicinchoninic acid (BCA) protein assay (Pierce, USA). For the enzyme reaction, 90 μl of cell lysate (30 μg protein) was mixed with 10 μl of 10 mM L-DOPA solution in a 96-well plate. The mixture was incubated at 37°C for 1 h. The formation of dopachrome was quantified by measuring absorbance at 405 nm using a UV/visible spectrophotometer (SpectraMax M2, Molecular Devices). Tyrosinase activity was normalized to the total protein concentration.

### SDS-PAGE and Western Blot Analysis

After treatment, the cells were washed twice with cold PBS and lysed in RIPA buffer (Thermo Fisher Scientific, USA) supplemented with protease and phosphatase inhibitor cocktails (Sigma-Aldrich). The lysates were incubated on ice for 30 min with occasional vortexing and then clarified by centrifugation at 12,000 ×*g* for 15 min at 4°C. The supernatants were collected, and protein concentrations were determined using a Pierce BCA Protein Assay Kit (Thermo Fisher Scientific). Equal amounts of protein (30 μg) were mixed with 5× SDS sample buffer, boiled at 95°C for 5 min, and separated on 12% SDS-polyacrylamide gels. The resolved proteins were transferred to a nitrocellulose membrane (Thermo Fisher Scientific). Membranes were blocked with 5% non-fat dry milk in Tris-buffered saline containing 0.1% Tween-20 (TBST) for 1 h at room temperature to prevent nonspecific binding. The membranes were incubated overnight at 4°C with primary antibodies diluted in TBST containing 5% BSA. After washing three times with TBST, membranes were incubated with horseradish peroxidase (HRP)-conjugated secondary antibodies for 1 h at room temperature. Protein bands were visualized using an ECL Select Western Blotting Detection Reagent (cat. no. RPN2232; Cytiva, USA) and detected with an LI-COR C-DiGit Blot Scanner (LI-COR Biosciences, USA). Densitometric analysis was performed using the UN-SCAN-IT gel analysis software, version 5.1 (Silk Scientific Inc., USA), and target protein levels were normalized to β-actin as the loading control.

### Statistical Analysis

All experiments were repeated at least three times. Statistical analysis was performed using GraphPad Prism version 5.0 (GraphPad Software, Inc.), and data are presented as the mean ± standard deviation. Data was analyzed using one-way analysis of variance followed by Bonferroni's post hoc test. *P* < 0.05 was considered to indicate a statistically significant difference.

## Results

### LSM Enhances Extracellular and Intracellular Melanin Production in B16F10 Cells

**Fig. 2:**
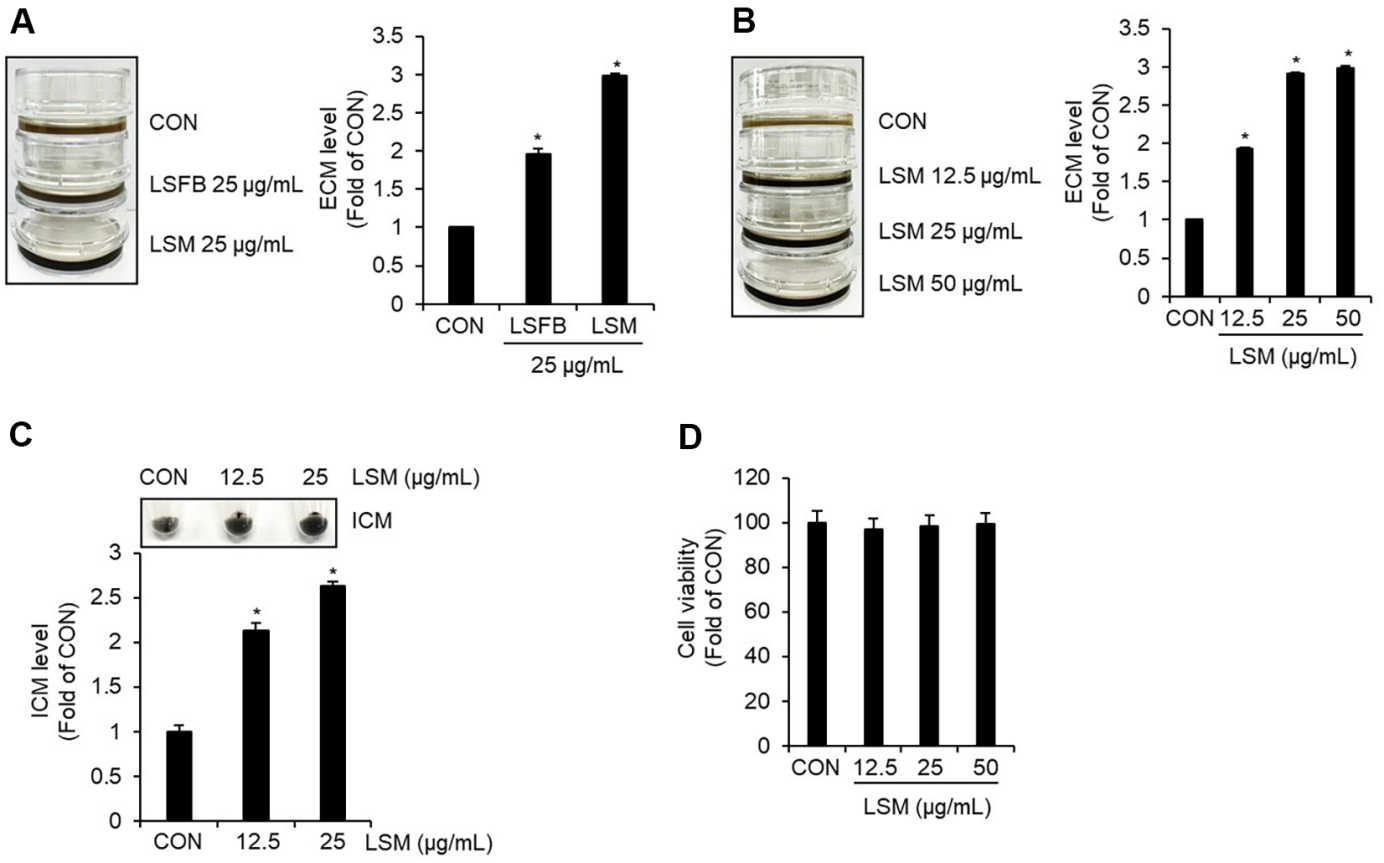
Effects of *Laetiporus sulphureus* extracts on melanin production and cell viability in B16F10 cells. B16F10 cells were treated with LSFB or LSM for 48 h. Extracellular and intracellular melanin levels were quantified by measuring absorbance at 405 nm. (**A**) Comparison of ECM content between LSFB- and LSM-treated cells. (**B**) ECM levels following treatment with LSM (12.5-50 μg/ml). (**C**) ICM levels following treatment with LSM (12.5-50 μg/ml). (**D**) Effects of LSM (12.5-50 μg/ml) on B16F10 cell viability as determined by MTT assay. *P < 0.05 vs. CON (untreated group).

To evaluate the melanogenic potential of *L. sulphureus*, LSFB and LSM were compared for their effects on ECM content in B16F10 cells. The results showed that LSM significantly increased ECM levels compared to LSFB (Fig. 2A). Based on this finding, subsequent experiments were conducted using LSM. Treatment with LSM at concentrations ranging from 12.5 to 50 μg/ml led to a dose-dependent increase in ECM content (Fig. 2B). In addition, ICM levels were also elevated in a dose-dependent manner, with significant increases observed at 12.5 and 25 μg/ml of LSM (Fig. 2C). To exclude the possibility that the LSM-induced increase in melanin production was a secondary consequence of cytotoxicity, cell viability was assessed using an MTT assay under the same treatment conditions. As shown in Fig. 2D, LSM did not significantly affect the viability of B16F10 cells at concentrations of 12.5, 25, and 50 μg/ml, indicating that the LSM-induced enhancement of melanin production was not attributable to reduced cell survival.

### LSM Upregulates Melanogenesis-Associated Protein Expression and Tyrosinase Activity in B16F10 Cells

**Fig. 3:**
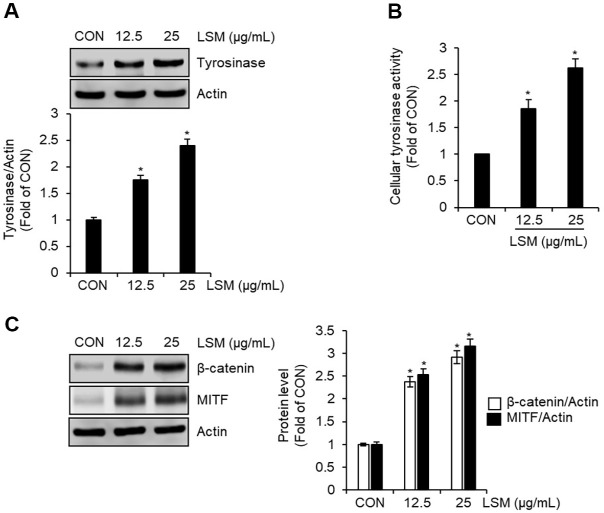
Effects of LSM on melanogenesis-associated proteins and tyrosinase activity in B16F10 cells. B16F10 cells were treated with LSM (12.5 and 25 μg/ml) for 48 h, and protein expression and enzymatic activity were evaluated. (**A**) Western blot analysis showing a dose-dependent increase in tyrosinase protein expression. (**B**) Cellular tyrosinase activity following LSM treatment, determined using L-DOPA as a substrate. (**C**) Western blot analysis of β-catenin and MITF protein levels, demonstrating their upregulation in response to LSM treatment. *P < 0.05 vs CON (untreated group).

Following the observation that LSM enhances both extracellular and intracellular melanin production in B16F10 cells, we further investigated whether this effect is associated with changes in melanogenesis-related signaling components. Western blot analysis revealed that LSM treatment (12.5 and 25 μg/ml) led to a dose-dependent increase in tyrosinase protein levels (Fig. 3A). Consistently, cellular tyrosinase activity was also elevated in a dose-dependent manner following LSM exposure (Fig. 3B). In addition, LSM treatment resulted in marked upregulation of β-catenin and MITF protein levels, both of which are known upstream regulators of tyrosinase transcription (Fig. 3C). These results suggest that LSM promotes melanogenesis at both the transcriptional and enzymatic levels by activating core regulatory pathways.

### LSM-Induced Melanogenesis Is Mediated by p38 MAPK Activation in B16F10 Cells

**Fig. 4:**
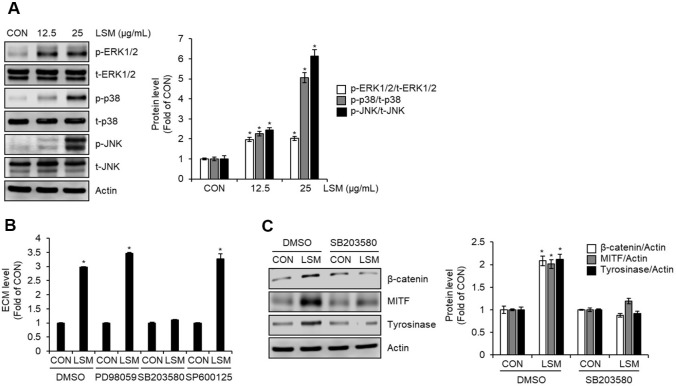
Involvement of MAPK pathways in LSM-induced melanogenesis in B16F10 cells. (**A**) Western blot analysis showing dose-dependent phosphorylation of ERK1/2, p38, and JNK in B16F10 cells treated with LSM. B16F10 cells were treated with LSM (12.5 and 25 μg/ml) for 48 h. The protein levels of p-ERK1/2, ERK1/2, p-p38, p38, p-JNK, and JNK were analyzed using Western blot analysis. (**B**) Effects of specific MAPK inhibitors on LSM-mediated ECM production. B16F10 cells were pretreated with PD98059 (10 μM, ERK1/2 inhibitor), SB203580 (10 μM, p38 inhibitor), or SP600125 (10 μM, JNK inhibitor) for 2 h and then exposed to LSM (25 μg/ml) for 48 h. Only p38 inhibition abolished the LSM-induced increase in ECM levels. (**C**) Western blot analysis showing that inhibition of p38 with SB203580 (10 μM) suppressed the LSM-induced upregulation of β-catenin, MITF, and tyrosinase protein expression. B16F10 cells were pretreated with SB203580 (10 μM, p38 inhibitor) for 2 h and then exposed to LSM (25 μg/ml) for 48 h. The protein levels of β-catenin, MITF, and tyrosinase were analyzed using Western blot analysis. *P < 0.05 vs CON (untreated group).

To elucidate the signaling mechanisms underlying LSM-induced melanogenesis, we investigated the activation of MAPK pathways. Western blot analysis demonstrated that LSM treatment (12.5 and 25 μg/ml) led to a dose-dependent increase in the phosphorylation of ERK1/2, p38, and JNK in B16F10 cells (Fig. 4A). To determine the functional relevance of each MAPK component, specific inhibitors were employed. The p38 inhibition by SB203580 (10 μM) abolished the LSM (25 μg/ml)-induced increase in extracellular melanin production, whereas the inhibition of ERK1/2 by PD98059 (10 μM) and JNK by SP600125 (10 μM) had no significant effect on LSM-induced increase in ECM production (Fig. 4B). Furthermore, inhibition of p38 with SB203580 (10 μM) suppressed the LSM (25 μg/ml)-induced upregulation of β-catenin, MITF, and tyrosinase protein expression (Fig. 4C). These findings indicate that the p38 MAPK pathway plays a central role in mediating the pro-melanogenic effects of LSM, likely through regulation of β-catenin/MITF signaling and downstream melanogenic enzymes.

### LSM-Induced Upregulation of Rho Family Proteins Is Mediated by p38 MAPK Activation in B16F10 Cells

**Fig. 5:**
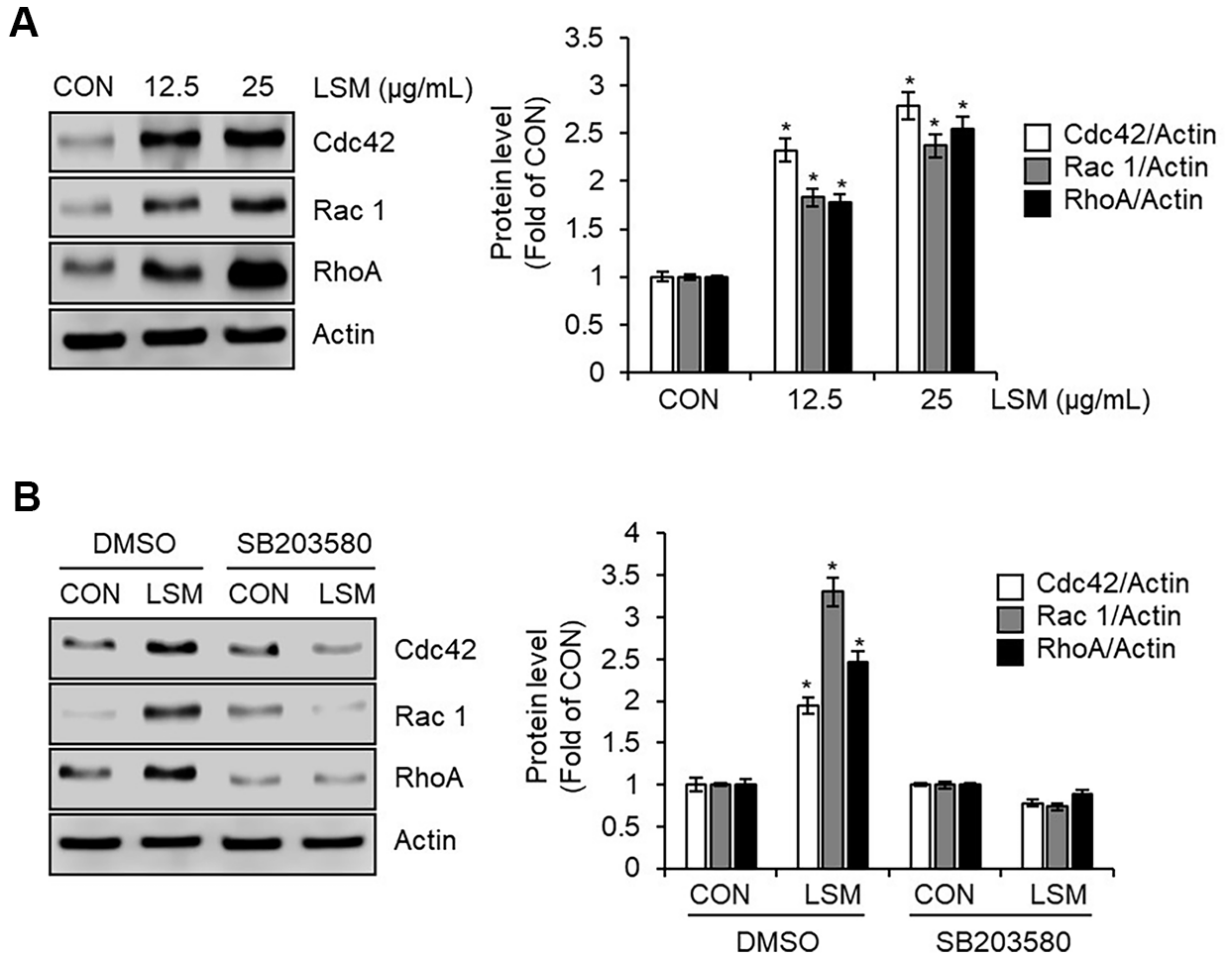
Effects of LSM on Rho family GTPase expression in B16F10 cells. (**A**) Western blot analysis showing that treatment with LSM dose-dependently increased the protein expression of Cdc42, Rac1, and RhoA. B16F10 cells were treated with LSM (12.5 and 25 μg/ml) for 48 h. The protein levels of Cdc42, Rac1, and RhoA were analyzed using Western blot analysis. (**B**) Effects of p38 inhibition on LSM-induced Rho GTPase expression. B16F10 Cells were pretreated with SB203580 (10 μM, p38 inhibitor) for 2 h, followed by LSM treatment (25 μg/ml) for 48 h. The protein levels of Cdc42, Rac1, and RhoA were analyzed using Western blot analysis. p38 inhibition abolished the LSM-induced upregulation of Cdc42, Rac1, and RhoA. *P < 0.05 vs CON (untreated group).

To further investigate the mechanisms by which LSM promotes melanocyte dendricity and melanosome transport, we examined the expression levels of key small Rho GTPases involved in cytoskeletal remodeling. Western blot analysis revealed that treatment with LSM (12.5 and 25 μg/ml) dose-dependently increased the protein expression of Cdc42, Rac1, and RhoA in B16F10 cells (Fig. 5A). Importantly, pretreatment with the p38 inhibition by SB203580 (10 μM) abolished the LSM (25 μg/ml)-induced upregulation of Cdc42, Rac1, and RhoA (Fig. 5B), indicating that activation of p38 is required for LSM-mediated Rho GTPase expression. These results suggest that p38 MAPK signaling is not only essential for LSM-induced melanogenic protein expression but also regulates cytoskeletal remodeling through modulation of Rho family GTPases.

### LSM Enhances ECM and ICM Production in HEMn-MP Cells

**Fig. 6:**
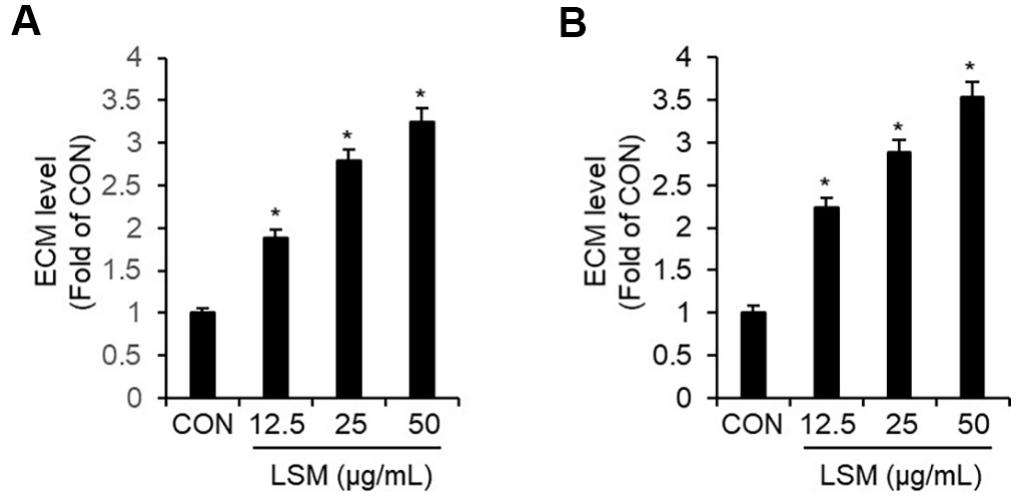
Effects of LSM on ECM and ICM production in human epidermal melanocytes (HEMn-MP). HEMn-MP cells were treated with LSM (12.5, 25, and 50 μg/ml) for 48 h, and melanin levels were quantified by measuring absorbance at 405 nm. (**A**) ECM levels following treatment with LSM (12.5-50 μg/ml). (**B**) ICM levels following treatment with LSM (12.5- 50 μg/ml). *P < 0.05 vs. CON (untreated group).

To determine whether the melanogenesis-enhancing effect of LSM observed in B16F10 cells could be reproduced in human melanocytes, we next examined its effect on ECM and ICM content in human epidermal melanocytes, HEMn-MP cells. As shown in Fig. 6A, LSM treatment increased ECM levels in a concentration-dependent manner, reaching 1.89-, 2.79-, and 3.25-fold of the untreated control (CON) at 12.5, 25, and 50 μg/ml, respectively. A similar trend was observed for ICM: LSM elevated ICM to 2.24-, 2.89-, and 3.54-fold of CON at 12.5, 25, and 50 μg/ml, respectively (Fig. 6B). These findings indicate that LSM robustly enhances both ECM and ICM production in human epidermal melanocytes, supporting the translational relevance of the melanogenic effects originally observed in B16F10 cells.

## Discussion

In this study, we demonstrated for the first time that LSM significantly enhances the production of both ECM and ICM in B16F10 cells. This melanogenic effect was accompanied by upregulation of tyrosinase expression and activity, as well as increased levels of MITF and β-catenin. Mechanistically, LSM activated the MAPK signaling cascade, with p38 MAPK playing a central role in regulating downstream melanogenesis-related factors. Interestingly, the expression of Rho family GTPases (Cdc42, Rac1, and RhoA), which are involved in cytoskeletal dynamics and melanosome trafficking, was also upregulated by LSM in a p38-dependent manner. Furthermore, LSM increased both ECM and ICM in human epidermal melanocytes (HEMn-MP) in a concentration-dependent manner, supporting that the melanogenesis-enhancing effects observed in B16F10 cells are reproducible in human melanocytes and may be of physiological relevance.

In this study, a comparison between LSFB and LSM revealed that LSM significantly increased ECM content in B16F10 cells. This difference may be attributed to the distinct metabolic characteristics of mycelia and fruiting bodies. Generally, mycelia are enriched in secondary metabolites such as polysaccharides, phenolic compounds, terpenoids, and amino acid precursors during their growth phase, whereas fruiting bodies tend to accumulate structural components required for reproductive development [15]. Therefore, the mycelial extract is more likely to contain higher concentrations of bioactive compounds that directly or indirectly stimulate melanogenesis, supporting the observed increase in ECM. These findings suggest that mycelial extracts may serve as more suitable resources than fruiting bodies for studying and utilizing melanogenesis-promoting activity. Treatment with LSM at concentrations of 12.5-50 μg/ml resulted in a clear dose-dependent increase in ECM, indicating that the melanogenic activity of the extract is positively correlated with its concentration. The concurrent elevation of intracellular melanin (ICM), particularly the significant increases observed at 12.5 and 25 μg/ml, further supports the conclusion that LSM enhances melanin synthesis within cells as well as its subsequent secretion.

The results of this study demonstrate that LSM not only promotes melanin production but also activates key regulatory factors involved in melanogenesis in B16F10 cells. The dose-dependent increases in tyrosinase protein expression and enzymatic activity indicate that LSM directly reinforces the central enzymatic step of melanin synthesis. Furthermore, the pronounced upregulation of β-catenin and MITF is particularly noteworthy. β-catenin, as a pivotal mediator of the Wnt signaling pathway, translocates into the nucleus and induces MITF expression [16] while MITF is well recognized as a master transcription factor that directly regulates the transcription of tyrosinase and other melanogenic enzyme genes [17]. Therefore, the concurrent induction of β-catenin and MITF by LSM is closely linked to the fundamental promotion of melanogenesis at the transcriptional level. Taken together, these findings suggest that LSM enhances melanogenesis not only through enzymatic regulation by increasing tyrosinase activity but also through transcriptional regulation via the β-catenin/MITF axis, thereby strongly promoting pigmentation. This comprehensive activation of melanogenesis-related pathways highlights the potential of LSM as a natural pigmentation-promoting agent.

In this study, LSM was found to induce the phosphorylation of ERK1/2, p38, and JNK in B16F10 cells; however, functional analysis revealed that p38 plays a pivotal role in mediating its pro-melanogenic effects. Inhibition of p38 markedly suppressed the LSM-induced increase in ECM as well as the upregulation of β-catenin, MITF, and tyrosinase protein expression, indicating that p38 functions as a central axis in the regulation of melanogenesis. This is consistent with previous reports showing that p38 MAPK enhances MITF expression and increases tyrosinase activity, thereby promoting melanin synthesis [18, 19]. In contrast, although ERK1/2 and JNK activation were observed, inhibition of these kinases had little effect on the LSM-induced increase in ECM. This finding aligns with previous studies indicating that ERK1/2 and JNK exert context-dependent roles in melanogenesis. For instance, transient activation of ERK1/2 can promote melanogenesis, whereas sustained activation is known to induce MITF degradation and consequently suppress melanin synthesis [20]. Similarly, JNK has been reported to exert either positive or negative effects on melanogenesis depending on the specific cellular context and stimuli [21]. Thus, the phosphorylation of ERK1/2 and JNK observed in this study may represent general cellular responses to LSM rather than direct mediators of melanogenesis. Taken together, these results demonstrate that among the simultaneous activation of ERK1/2, p38, and JNK, the pro-melanogenic effect of LSM critically depends on the p38 MAPK pathway. This finding supports the conclusion that LSM promotes pigmentation by regulating the β-catenin/MITF axis and downstream melanogenic enzymes, while ERK1/2 and JNK likely play auxiliary or nonspecific roles.

In this study, we found that LSM increased the expression of Rho family proteins, including Cdc42, Rac1, and RhoA, in B16F10 cells, and that this effect was dependent on the activation of p38 MAPK. Rho GTPases are well-established regulators of cytoskeletal remodeling, controlling actin filament dynamics, dendrite extension, and intracellular transport processes [22, 23]. In melanocytes, the activities of Cdc42, Rac1, and RhoA are particularly essential for dendrite formation and the transport of melanosomes to the cell periphery [7, 24, 25], which ultimately facilitates the transfer of melanin to keratinocytes. The observation that inhibition of p38 completely abolished the LSM-induced upregulation of Rho family proteins indicates that p38 signaling serves as a central pathway regulating both melanin synthesis and pigment distribution. This is consistent with previous findings showing that p38 MAPK mediates cytoskeletal reorganization through regulation of Rho family proteins in response to cellular stress [26]. Therefore, LSM appears to promote melanogenesis not only via the MITF/tyrosinase-dependent pathway but also by activating Rho GTPases to enhance the morphological and functional maturation of melanocytes.

Furthermore, we extended our observations to human epidermal melanocytes (HEMn-MP) to assess the translational relevance of LSM-induced melanogenesis. Consistent with the findings in B16F10 cells, LSM markedly increased both ECM and ICM levels in HEMn-MP in a concentration-dependent manner. Notably, the magnitude of melanin induction in human melanocytes was comparable to, or even greater than, that observed in B16F10 cells, with up to ~3-fold increases in both extracellular and intracellular melanin at the highest concentration of LSM. These results indicate that the melanogenesis-enhancing effect of LSM is not restricted to a murine melanoma cell line but is preserved in normal human melanocytes, supporting the physiological relevance of LSM as a pigmentation-promoting agent. Taken together, our findings provide strong evidence that LSM promotes pigmentation by simultaneously regulating melanogenic protein expression and Rho GTPase-mediated cytoskeletal remodeling through p38 activation. This dual action enhances not only melanin synthesis but also its effective distribution, supporting the potential of LSM as a promising candidate for pigmentation-related therapeutic applications.

## Conclusion

In this study, we demonstrated that LSM significantly promotes melanogenesis in B16F10 cells and that this effect is dependent on the activation of p38. LSM not only increased the expression of key enzymes such as tyrosinase but also upregulated transcriptional regulators, including β-catenin and MITF. In addition, LSM activated Rho family proteins such as Cdc42, Rac1, and RhoA, thereby contributing to cytoskeletal remodeling and melanosome transport. These findings suggest that LSM promotes pigmentation through multifaceted actions encompassing enzymatic, transcriptional, and morphological regulation, supporting its potential as a therapeutic or functional agent for pigmentation-related applications.

Nevertheless, several limitations of this study should be acknowledged. First, the experiments were conducted using the murine melanoma cell line B16F10, and further validation is required to determine whether the same effects can be reproduced in *in vivo* skin models. Second, the specific bioactive compounds in LSM responsible for these biological activities were not identified, and chemical characterization remains necessary. Third, although we demonstrated that p38 MAPK plays a predominant role in LSM-induced melanogenesis among the MAPK family, the precise molecular mechanisms by which p38 MAPK signaling connects to Rho GTPase activity and the β-catenin/MITF axis require more detailed investigation. Fourth, we quantified only the total amounts of extracellular and intracellular melanin and did not distinguish between eumelanin and pheomelanin, so potential changes in the eumelanin/pheomelanin ratio in response to LSM treatment remain unknown. Fifth, signaling pathways outside the MAPK cascade were not systematically examined in this study, and their potential contribution to the melanogenic effects of LSM remains to be clarified. Therefore, future studies should validate the physiological effects of LSM using human melanocytes and animal models, isolate and identify the active compounds to establish structure–activity relationships, and elucidate not only the direct molecular interactions between p38 MAPK and its downstream regulators but also the involvement of non-MAPK signaling pathways at the mechanistic level, as well as perform detailed profiling of eumelanin and pheomelanin species to determine how LSM affects their relative balance. Such studies will further clarify the melanogenic effects of LSM and provide a crucial foundation for evaluating its clinical applicability.
